# Correction: Re-analysing Ebola spread in Sierra Leone: The importance of local social dynamics

**DOI:** 10.1371/journal.pone.0290847

**Published:** 2023-08-24

**Authors:** The PLOS ONE Staff

## Notice of Republication

This article was republished on July 31, 2023, to correct for errors in the Data Availability statement. The publisher apologizes for the errors. Please download this article again to view the correct version. The originally published, uncorrected article and the republished, corrected article are provided here for reference.

## Supporting information

S1 File Originally published, uncorrected article. (PDF)

S2 File Republished, corrected article. (PDF)
