# Supplementary material for: Re-analysing Ebola spread in Sierra Leone: The importance of local social dynamics
Source: PLoS One. 2020 Nov 5;15(11):e0234823. doi: 10.1371/journal.pone.0234823 (PMC7644078; doi:10.1371/journal.pone.0234823)
Supplement: S1 Village — (DOCX) [file pone.0234823.s001.docx]

**S1 Village**

**S1A Village (7^th^October, 2016, edited PR 10/4/2019)**

**Section: [deleted] Chiefdom: Kori District: Moyamba**

| **SN** | **NAMES** | **AGE** | **SEX** | **POSITION** | **OCCUPATION** |
| --- | --- | --- | --- | --- | --- |
| 1 | JL | 40 | M | Asst. Section Chief | Farmer |
| 2 | KY | 70 | M | Town Chief | Farmer |
| 3 | YD | 70 | M | Town Speaker | Farmer |
| 4 | JL | 55 | M | Elder | Farmer |
| 5 | JL | 22 | M | Student | Student |
| 6 | BN | 54 | M | Teacher | Farmer |
| 7 | MS | 26 | M | Youth | Farmer |
| 8 | HK | 58 | F | Elder | Farmer |

(The number against the answers are taken from the table above to identify those who spoke)

**Q. How and when did you hear about Ebola?**

1- We heard of this Ebola sick[ness] from Kailahun in 2014, but we had declaration of it in this village in July, 2014. Our town secretary can explain more but he is not around.

**Q. How would you describe Ebola to your unborn child?**

1- I will tell my child that the sick[ness] is an airborne disease and can also be contacted by eating bats and bush animals like monkeys. The signs are excess vomiting, red eyes, weakness and body rash. The precaution that we were told is to avoid any sick person and [that we] should not greet or shake hands with anyone irrespective of their health status, also anyone found [with] such signs should be taken to the hospital or [we should] call the nearest health post for swabbing, and if the person is positive [they] then should be taken to a special place till 21 days.

However, the real thing that cause[d] death in our village is the excessive spray[ing] of chlorine. There was a disease surveillance tracer from F and M, and later BN [No. 6, see above] was appointed to act as the contact tracer for this village.

3- We heard of the Ebola since [DATE MISSING?] and is called in Mende *bondawote* [family turn round]. If somebody contacts the sickness, you are completely abandon[ed] to die.

**Q. What is your experience about touching?**

3- We heard it from the Government that we should stay away from touching any sick person and if you do, then you are a carrier of the sick[ness] as well, and there is no treatment for the sick. The message was so intense that we have to be afraid of the sick, and that makes us to abide by the Government laws.

**Q. Do you believe the Government that touching and shaking of hands with people was a bad idea?**

2- It was much doubt[ed by] us as [not] all the signs told us by the government were seen on any sick person that died in this community, like blood coming out of mouth, ear, temperature high, weakness or body rash. So how can you tell whether it was Ebola that led to their death? It was due to these doubts that JL was invited to Taiama court barrie [to answer the charge] that he was passing the message round that there was no Ebola but all are lies. [On another occasion JL gave us an account of his trial and how he rebutted the charge. Basically, his defence was “why would any educated person take any notice of anything that an illiterate village chief said or thought, so how can I be guilty of spreading false information?”

AY [female] was the first person whose swab was collected for test by Mr. G [CHO], [and she] later died in the evening. After her death, the result came out positive according to the CHO, followed by [the deaths of] TB [female] and NM [female], [leading] the village to be quarantined for 21 days. Excess chlorine led to much sickness that people could not stand. In all we had 9 deaths.

2 Soldiers, 2 OSDs and 1 police man were sent to guard the town, [so] that no one can move around and out of the town. One of the soldiers, called N, asked whether it was really Ebola that was affecting the community because everyone is doing fine. In fact, the security that was supposed to have been [in place] for 21 days lasted for a month and the ODS had want[ed] to stay even after the quarantine (18^th^ August, 2014) period, due to the way they were encouraged by the community.

**Q. How fast did you learn that Ebola was peaking?**

2- Due to the touching that was restricted, [this] caused lots of deaths, as many people got sick [of other diseases] and were not cared for, [for] fear of contacting the sick, no treatment, [and] no food or water was given to anyone, even by a family member.

6- The dress of the burial team brought shock and fear in the community [and this] led to plenty sick[ness], especially the way the chlorine was administered in the village and on themselves, while no family member was allowed to go close [to the body] for any funeral or burial.

5- We saw the burial team as having a hidden agenda, because they go in [to] collect blood from the corpse, [that is] placed into a plastic bag, and the [burial team] goes alone as a team for burial.

**Q. what would you have preferred partners involved to do about burials?**

3- They [sh]ould have train[ed] more burial teams at sectional level, and [members ought to] have come direct from this village, for example, as we are the first people, according to the Government, that had the sick[ness in this area]. Provide enough PPEs.

7- Provide food [for] us, as [we] were abandon[ed]. Later 7 persons to a bag of rice w[as] given. Nobody ever tried to bring any development to us as a first place of contact in Moyamba District, [it was] as if they had plans for us all to die.

**Q. What can you say about the dispensation of the Medical team?**

1- A team from Moyamba came to collect [a] blood sample but was asked to return by JL because there was no call to confirm their coming, not even by the Paramount Chief or the CHO. The report went to the CHO [who] then he decided to come with the team. When he came, he stopped by the river and ask the boys to go, but JL asked that he himself come and do the blood sample collection, and that is what happened. The late MS was part of the team that went for the sample collection but did not go close to the house were AY was, [the woman] whose blood was to be collected.

3- My doubt was also that every sick person needs blood but [I worried that] all [the blood needed to] sustain the sick [person would be] remove[d] and the person will not stand it, but [will] die.

**Q. What was the community reaction?**

8- Most people ran to their farms as a place of rescue [safety] for fear of not touching each other. Even the nurse (RY) ran away from our Health Post in this community, [but later she] came for food supply.

5- Continuous prayers were offered in our individual home[s] because we thought God has forsaken us.

**But what kind of settled understanding then emerged?**

4- We keep away from everyone, as we got it from the chiefdom that it is a law. So we abide[d] by it as a caution to us, as the river was at [its] peak [August].

**So how did people react to this?**

1**-** People ran to their farms and stay[ed] in the bush. No one goes close to any sick person. No giving [of] water to drink to any patient, no drugs w[ere] available. Even the nurse ran away from the PHU in this village but when the food supply came the nurse came to collect her share. Prayers and ceremony.

**What pattern did you begin to see?**

3**-** Prayers and sacrifices in our individual homes made the sick [to] lessen and even [to] come to an end.

**Did you discuss this caution among yourself?**

3**-** We advise[d] people to be careful and keep washing their hands. Some decided to go to their farm houses. Most people learnt by experience.

**Did you take any protective activity based on the pattern observed?**

3**-** Touching was forbidden, people ran to their farms. Most of those who got sick were recovering when they stop[ped] touching each other.

**Who took the protective measures?**

3**-** The PC and some medical team called the acting section chief (JL) to explain what should be done; and [that] they should report all sickness, and not touch, [and that] washing of hands is a matter of most importance; we were given two buckets with soap around 14^th^ June 2015 [outbreak earlier said to be July]. The chiefs in the chiefdom made the laws more active, that anyone that [went] against [them] will pay Le. 500,000 as a fine.

Quarantine – [started] 18^th^ August 2015 [2014], 2 OSD’s, 1 Police, 2 Soldiers. [On] Wednesday they slept in Village 1C [on the main road] as they [had] heard that there was no one [left] alive in the village. Then on Thursday they decided to come [here] and they stayed up to a month because we took care of them, as they were our brothers. They were expected to spend 21 days but they were here up to a month.

**On what Ebola was and why it struck [in] the way it did?**

4- One thing we need to know is to obey the leaders in the country. My doubt is the blood oozing out of the sick patient; [this was] not seen or realized.

5- (JL) I believed that Ebola was a severe sickness and [that] it is real, as in the household of Pa S, [that] was where the sickness start[ed] and hit the direct people in the same house [infection].

6- I believe [Ebola is real] because I witness[ed] someone that had Ebola, and all those that touch[ed] him died after his death.

**What [do] have you to say about the claim that someone came into this village with this sickness?**

4- Mr. JK, medical assistant at the health centre here, told us that Mr. S came here with the sick[ness] because he came from Kenema after going through several checks along the way to [here] at checkpoints.

[Group confirmed that nine people died in this village, two people were swabbed, only AY was tested and found positive for Ebola].

?- We are not happy because Ebola struck here first, as our name was “[Village 1A]-Ebola” but no help ever came our way, despite the misery we went through, maybe due to the doubts we raise[d] about the sick[ness] and against witnessing the signs of Ebola.

?- Plenty [of] promises are made but no one [has] ever fulfilled their promise, be it in food, medicine and tents. We later heard that the tents (mosquito nets) are meant for pregnant women; as most of us lost our wives and we don’t have [a] plan of marrying any woman or [being] able to impregnate a woman even, so we count ourselves out [of the distribution] totally.

**Can you give us a brief background about Pa Sandy [index case]?**

? - All those who washed and buried A and Pa S are alive. MS carried Pa S from the river into his house [here]. Even the bike man that took him from Village 1C is still alive. The person that shaved him (Mr. F) is alive also. [It is only] his niece, AY, that died.

? - AS`s motherland is here; [he was] aged 75 yrs and his fatherland is Waima, the village [where they] broke the bridge.

? - He was all these years in KK, Kenema District (Lower Bambara chiefdom) as a diamond miner

? - He had plenty [of] children, including those that help[ed] in the mining.

? - Pa S visit[ed] this village [at unpredictable times] after every 2 years.

? - He came for his uncle (Pa L)’s funeral at here, [and] after the 40^th^ day [ceremony] he returned. It was not up to a year [that] he last left the village and came here on the 9^th^ July 2014

? - GS who was in Kenema and [is] a son of Pa S. got sick and died at the Kenema Government Hospital.

? - He had a heart attack at the gate when the ambulance took his son for burial without seein[g] his corpse.

? - T. the wife of late GS is alive at Bai Largo

? - Pa S.`s other son in Kenema, BS, died and was a proprietor of xxxx Academy Kenema. M. [female] was part of the team that came with Pa S. to Village 1C and is alive; she lives at xxxx section in Kenema. MS also is alive; [he is the person] who took him [Pa S] from the car and held him on the motorbike to the river.

? - He [Pa S] was excessively sweating and was speechless

HK [female] - I went to receive Pa S from the river and held his hands till MS took him on his back. He walk[ed] in and sat on the bed alone. He never had frequent stool, [or] vomiting, [and] no sign of Ebola. He stayed here up to two weeks before his death. [During that time] He was speechless. It was during Ramadan period in July that he got worse and died. He mostly used to sleep in the mosque and his room. AF the barber that shaved Pa S is still alive. Late MK [female] had her sick[ness] from Freetown around June 30^th^ 2014, not from this village, because she was not in any of the funerals.

**S1B Village. Visit to Village 1B February 18^th^ 2017 [PR and GAM]**

This is the village from which relatives and in-laws came to sympathise with the first woman to contract Ebola in Village 1A. Some of these visitors also caught the disease. They know it was Ebola because three of them were taken to Moyamba Holding Centre where two died, but not before the third, an old man, who was Ebola-negative and later discharged, was informed that the tests of his two fellow villagers were positive for Ebola. The old man returned to the village, where he told his story to others. At the outset the three sick people were carried by canoe across the river and then taken by okada bikes from a village on the opposite bank to village 1C on the main road. On 18^th^ February 2017 we crossed from the same point to Village 1B. We were surprised to find at this time of year the river is shallow enough at this point to be waded, even though it is more than 100 m wide. Village 1B is small [about 10 houses] and lies on a bush track running south along the left bank of the river from Village 1A to Village W. There is a high stick bridge across a ravine about 2-3 km south of Village 1B. From this bridge the path continues southwards to the larger village of W., where there is a PHU. On hearing that there was suspected Ebola in Village 1B two youth leaders in Village W. decided to cut down the bridge, to prevent sick people reaching their PHU, because by this stage they were aware that the sickness would spread to unprotected PHU staff. This was in August or September 2014, and effectively cut off Villages 1A and 1B from the rest of the chiefdom during the Ebola outbreak. Villagers in Villages 1A and 1B were very upset, fearing they had been abandoned by the world. Some farming families in Village W were also angry because they could no longer reach their ripening rice farms north of the ravine. Discussions in Village 1B confirmed survey data collected in both villages in November 2014 relating to Ebola spread. Afterwards, we walked up to village 1A through the gallery forest and crossed the river in the canoe of Village 1A.

**S1C Village - Kori Chiefdom, Moyamba District (27^th^ September, 2016, edited PR 11/04/2019)**

**Focus Group Discussion**

**Attendance:**

1. AS, Town Chief, 52 yrs, male, Muslim, trader, farmer.
2. FS, male, elder
3. JSD, male, elder
4. JBT, male, teacher
5. FT, female, Youth Leader
6. BB, female, assistant Youth Leader
7. JM, male, Teacher
8. KB, female, Elder
9. MA, male, elder, 62 yr, Muslim
10. MT, male, elder, Imam

**Q. What can you to tell us about the past Ebola sickness that affect our country in general?**

FS This is a sickness that collects [infects?] all members of a family or more.

**Q. When actually did you hear about the sick[ness] (Ebola**)

JBT We started hearing of Ebola early May 2014 in Kailahun.

**Q. Did Ebola affect any one of you directly or your community?**

AS Yes, it affected me and the entire village, especially my very [good] friend [name not mentioned] and his family. On the 11^th^ September 2014, the wife (MS) of my friend (DS) died [as] a confirmed carrier of Ebola at the Medical Centre in Taiama, Chiefdom Headquarter of Kori, followed by her husband (DS).

JSD On the highway leading to Bo and Moyamba Junction, MS was the first suspected carrier of the Ebola virus but was not confirmed of where she got it. The late woman got sick and my wife suspected her and even helped her to the medical centre on a motorbike, were she later died.

**Q. Do we have anyone staying in the house of the late S family of Village 1C that can actually give us some summary of what went on?**

JSD, male, 45 yrs, was in the same compound [as] the late S. Family)

According to report we had, there was one Pa S who travelled from Kenema to Village 1A but entered this community [Village 1C] as it is the major entry point leading to Village 1A which is about six miles from us and is beyond the river. Village 1A is Pa S`s home but he spent most of his time in Kenema staying with his children. One of his children got sick of the disease and he was the main person caring for the child who later died in Kenema. It was due to this that the Kenema District Heath Management Team (DHMT) went for him as he was also manifesting the signs of Ebola. The mindset of the family members was that if their father goes to the [Ebola] centre [Lassa Fever Isolation Ward at KGH], he will not come [out] again, as anyone who goes to the centre will be no more, as it happened with their sister. So, they decided to hide with their father when Kenema was under quarantine [and come] down to Village 1C, the last point that vehicles stop [for passengers wanting to reach Village 1A]. They even went as far as the school, so that many people will not be aware of their coming in the hired taxi. Though it was late, every transportation facility was [still] in service [including] the motorbike that was to take him to the river and the canoe that will be used to cross over to the village. However, late DS was aware of all this movement without alarm, as Pa S from Kenema was his father-in-law, who happened to be late Mrs. MS`s uncle [MS’s father’s brother?].

Meanwhile, late Pa S from Kenema was received by one AY [female], a relation of his in Village 1A, and she took care of him before he finally passed [away].

Late MS, seen as a contact person of the carrier of the disease, went with the DHMT to Village 1A to sensitise her family to be careful of the sickness, and decided to take her mother [from Village 1A] [back] with her to Village 1C, while the DHMT went to collect blood from AY (who got sick after the death of Pa S from Kenema) to test whether it was Ebola or not. After AY`s death the report came in two days that she [had] died of Ebola. Most people say MS came [to Village 1C] with the sick[ness] but according to late MS, she did not even step her foot [inside] the late AY`s house, neither [did she] partake of any activity with the DHMT [delegation], nor did she visit any other house in the village except [that of] her mother because she was actually afraid of the sick[ness].

When MS died at the health centre in Taiama, the CHO (Mr G) claimed she got the virus from her visit made [to Village 1A] on th[e] same day the DHMT went for the collection of [a] swab [or blood sample?] from the late AY.

**Q. What can you tell us about the late MS, the first suspect in Village 1C?**

JSD Late MS was the wife of the late DS. The late Pa S from Kenema was MS`s uncle from Village 1A, which is her motherland. MS went with the swabbers from Moyamba to take the blood sample of AY [who became] sick after the death of Pa S from Kenema. MS told me that her purpose of going into the community that very day was to sensitise her family about the disease and [that] they should be careful with their contact and [she then] decided to come [back to Village 1C] with her mother. She [MS] got sick later and it was detected by my wife that MS seemed not to be OK. In the interim, MS decided to move from her matrimonial bedroom and join her mother (MT, now returned to Village 1A, aged 65) in the next room of the same house, and was with the mother sleeping on the same bed till she was taken for treatment, but as we speak the mother of the late MS is still alive and healthy. My wife, who is also still OK, went with her on a motorbike to the Community Health Centre at Taiama. On their arrival MS [was about to] die; that was why he[r] corpse was not taken in for treatment but was placed on a long bench outside the PHU. She was there until the Burial Team was called and came to bury [her] at the Taiama Cemetery.

**Q. In similar vein, can you tell us about the late DS?**

AS. Late DS was a personal friend [of mine] and I used to spend [a] lot of time with him at his business centre, as he was a business-man as well. This man had [a] compound in this community and even tenants like JD and [his] family and many others. To start with, when Pa S was brought in this town to cross [to Village 1A], this person [DS] was the only man aware of all the plans. He even went to the school building to see the sick man and share greetings with the children, as a custom [in which] one needs to pay respect to elders, [not to mention that] Pa S [had] was his father-in-law.

The late DS went for the burial of Pa S [in Village 1A] and returned to Village 1C. During the fortieth day ceremony he again went for the ceremony as a family member, but the wife did not attend any of the above-mentioned programmes.

Late DS used to complain of being sick [regularly], starting from the Muslim fast season.

On his return, he continued the same talk of not feeling OK. He was not actually feeling OK, but I cannot tell whether it was due to his sickness that the wife decided to transfer to her mother`s room or because she was not feeling OK too, [so] as to prevent her husband from contracting the sick[ness].

On the 18^th^ September, 2014, late DS was more serious[ly concerned] about his health and decided to go to BL for treatment [a village] on the highway to Moyamba Junction. Unfortunately for him the medical person in charge was not around and he (DS) decided to go down to Moyamba Junction for treatment. As God would have it, he [there] met the pharmacist at the junction for treatment but the pharmacist did a referral to late JB [the pharmacist?]. The late JB did the treatment and he [DS] returned to Village 1C. Two days later, he continued to complain of not [being] OK.

Meanwhile there was a contact tracer (RK, female) who stays in the same compound of the family of the S family, and to our greatest surprise we saw an ambulance from Moyamba [come] to collect some blood from DS, to test what had been the cause of his illness. The ambulance came and [stood] for about 10 minutes at the top end [of the] road leading towards Taiama and later turned to break at the house. It was during this movement that RK (Contact Tracer) ran to my house to [get me to] be a representative to approve their work of collecting the blood sample and [that] they are not forcing anyone [in] do[ing] their work, even the patient. Unfortunately, I was not around then, but my brother represented me. As the ambulance team was talking, DS heard them and decided to call on them to come in and do the blood collection as he was fighting to make sure he is back on his feet. A day after, DS died on the (22^nd^ September, 2014). His death was noticed by his sister (NS from M. a village about 3 miles from Village 1C on the highway to Taiama) who later called her elder brother (BS) from the village of BL. When the brother came, he raised the alarm [concerning] DS`s death to the entire community and later called the CHO in Taiama. The CHO called for the burial team, who did not come till about 9 pm, and the burial was done that very night.

On the 24^th^ September 2014 test result came and confirm[ed] DS as Ebola positive. The CHO (Mr. G) declared that the house should be quarantined on the 25^th^ September 2014 for 21 days without demarcation or a tape, and during this [period] all the children refused eating for the loss of their parents (SS 13 years, MS 9 years, IS 6 years and MS 4 years), including NS and late MS`s mother (MT, 65 years) who later left after Sierra Leone was declared Ebola free. Days later IS got severely sick and died, the medical team never came around to treat them, but food was supplied. MS [the child] died after, followed by MS. However, SS is still alive; she is not a biological daughter of the late DS. SS is BS`s daughter from the village of BL. NS also left after the declaration of Sierra Leone free of Ebola [and is] now staying in Freetown. MT is in Village 1A.

In general, the community was surprised to realise that the sick[ness] just finished the entire DS family without touching or infecting anybody [else] no matter the closeness.

**Village 1C, part 2 (8^TH^ October 2015)**

RK (female) – contact tracer (CHW), Age: 50. Occupation: teacher.

My area of work was specifically in Village 1C

Looking out for some sicknesses like headache, joint and body pain, diarrhoea, vomiting, bleeding, high fever, red eye, cough.

Reported two cases in this community

**MS** – victim, married to DS, 35 years, housewife, petty trader.

I was staying with MS in the same compound, but not in the same room. She got sick early September. The husband took her to Taiama in [to] one of the house, not to the health centre. When she became worse and people got suspicious, then she was taken to the centre almost dead, and the day did not pass [before she died]. It was on the 11^th^ September 2014. I asked MS to go for treatment, but she refused. One night we heard that Pastor I. has asked that we all wash with salt water; that we all did, and it was after this [that] she became very sick and was carried on a motorbike with her friend ID, who is still alive.

After the salt wash, she complained of joint pains

I saw her urinating red, and with no stool

Husband (late DS) paid her way

I made follow–up with ID (wife of JSD) to know whether MS was directly taken to the health centre.

BK (daughter of RK) had some joint pain, after hitting her foot on a stump; she was also carried to [CHO] G to check and treat her. BK had Sickle Cell [anaemia] also; during her pains she started swelling after taking ORS, and was pregnant. My doubt about her then was [that] swelling is not a sign of Ebola.

**Q. How were the interactions with the S family?**

Interaction was among us, all be it adults [or] children

DS started showing signs of fever, diarrhoea and loss of appetite

DS went with NS his sister to BL for treatment at the health centre, where MK was the nurse in charge, but dead now.

**Q. How were you quarantined?**

We were isolated, not quarantined, so as to keep the family and all in the compound from reaching other people in this Village 1C for some time.

Quarantine – [there] is no access to others, and should have a demarcation tape.

Their [who?] daughter NS (5 years) died on the 19^th^ October 2014, by refusing to eat, followed by IS (9 years) who got fever RDT+ and died.

SS - who was DS`s second wife from M. village - came and stay[ed] with DS after MS`s death, with her daughter MS (3 years) [who also?] died.

**Q. How did the ambulance find its way into this community?**

I was given a phone by Mr G. from the Medical Team that had all emergency numbers, but the phone was stolen in this community. It was someone that called the ambulance and direct[ed] them to my place without my knowledge. The team met me and spoke with DS, who agreed for the collection of his blood sample.

The movement of the ambulance was due to mis-direction by someone, as they were moving from the beginning to the end of the village, and later located the house. [This was] a day after he [DS?] died.

DS died on the 22^nd^ September 2014

**Touching**

Chief AS realized Ebola after the death of DS and children, [that there should be] no touching any more.

Mr. Daniel [had] not known, even with MS hav[ing] no Idea of touching; we ha[d] been hearing of it, but DS’s death [wa]s the real evidence of Ebola, as the family got affected.

RK – Pa S 🡪 AY 🡪 B from Kenema.

RK – My brother (DK) from Freetown told me of the sick[ness] with chlorine before Pa S’s arrival. Implementation started

DK – ask[ed] for the chlorine from RK to secure his family

**Idea of how to care for the sick or the dead**

RK – the medical team should take care, and only the burial team should do the burial

DK – burial team treatment with the corpse

**Any doubts?**

DK – why [the] burial team touches corpses, without being a victim too. Ebola carries all symptoms of all sicknesses.

Chief – throwing corpses into the grave without prayers.

RK – blood oozing not common.

**Disagreement**

Chief – burial practices in the grave.

RK – improper handling of the corpse, not dressing or washing [it].

DK – PPE’s; they were not doing the burial, or do they want the sickness to become fearful?

RK – no community participation or family present. No empathy, sympathy, stigma [from?] carrying [body] bag with them inside.

- Ambulance
- Ineffective use of chlorine
- Signal [siren] of [the] ambulance

Chief – removal of blood without replacement. No caring for the sick.

**Agreement**

Chief – no touching, new sick[ness] accepted. [Agree we should] report about sick[ness] and death

**Settle understanding**

Abide by the local bye-laws.

Allow family members [to witness burial] with protective dressing [alongside?] the burial team

**Crucial Things**

Traditional head’s corpse became evil as someone’s shoe was left in the grave and about to be drawn [drowned?] in the river. Burial team decided to have [local?] members.

Village 1C – 57 households

1 household [only] experienced Ebola (S family)

**Q. What was the reaction of [the] other households?**

Community stigmatized them, used the street to enter mosque, less people for prayer.

Calling them “Ebola patients”

Immediate household neighbours moved away from the S’s. Not eating [with them] again [apart from?] the children.

I was in a doubt why is it that

RK avoided the family – no food sharing, no cloth[ing shared?], [no] touching

**Networking – chain**

Just one house, MS’s death was not agreed to be Ebola.

After the death of the three children the Village 1C community took it as real Ebola

**Protectives**

We were discussing:

- Washing of hands regularly
- No visitor allowed

**Q. Who undertook the action?**

- Middle of 2015, chiefs and local leaders with fines
- No security personnel were assigned here. 17^th^ September 2014. Isolation, not quarantine

**Q. On what Ebola was, and why it struck?**

- RK – Ebola is from the government, to have money
- To get blood groups and sell [them]
- The money was much, so we saw the government’s hands in it.
- The soap from the government on the three days lock-down was rejected.
- [We] stop[ped] movement to other houses
- [There was] prayer and hiding [of] sickness, even headache.

**S1D Village**

**GS, a pharmacist in Village D1**

Late GS was a popular drugs seller who owned a pharmacy at Village 1D. It is suggested by informants that DS of Village 1C came to GS for treatment, but GS refused to treat anyone at that moment and DS then decided to go to the pharmacy of MB for treatment. The wife of GS, HJ (age 37) survived the sickness after going through the discouragement of losing members of her household and suffering much marginalisation.

**Interview with HJ wife of late GS**

Q. Can you narrate exactly how the Ebola was contacted in your household?

My husband was a drug seller that many people came to for medicine. He used to have a pharmacy and was treating people, but he stopped all treatment before the emergence of Ebola, because he was not satisfied with the nurse [he employed]. That is the reason DS from Village 1C was refused treatment. At the time there was a radio jingle that all the sick people were to be taken to the health centres and not drug stores or pharmacies. As a man that cares for all, he decided to join the voluntary contact tracers and go out every day searching for the sick.

As a contact tracer, somebody (I., male) died in his room here at Village 1D while vomiting in a bucket. According to those who saw him, the man became stiff while his head was hanging over the bucket vomiting until he died. So, my husband voluntarily entered the room with a stick in his hand and pushed the corpse over so it would lay down well and removed the bucket with the same stick. The body was then collected by a burial team for burial.

Secondly, I was told of the funeral of one K., a person always with the late MB, that was done in the village of KF [see below]. I think he was more involved in that burial. But I can`t tell whether he touched the corpse. I. and K. were both close to the late MB.

Third point was that my husband used to travel on a motorbike and was even given a motorbike from the medical team from Moyamba for contact tracing. It might be the cold that affected him to be sick as he gets up early for work with the team so the sick will not escape to wherever.

It was not long into September that my husband got sick and complained of fever and was taken to the Moyamba Ebola holding centre and finally died on the 4^th^ October, 2014

**Interviews in Village KF, Fakuniya chiefdom (close to Village 1D)**

Attendance

AM (60 yrs) – male, Town Chief

KK (45 yrs) - female, survivor

GM (48yrs) - Elder

The directly affected houses that were quarantined.

1. The J. family house (it was a motorbike accident death but the family was quarantined)
2. The K. family house
3. Household of LL (female)
4. The household of WL (female)
5. SL household
6. KK (male) It was a cancer death case, but the family was also quarantined.

After the above houses were quarantined, the entire town went into quarantine in 2014.

Q. Were you having any taskforce here?

Chief AM - We did not have a body called “taskforce” but I was one of the contact tracers that was working in my community, with much pointing of hands (towards me) as a bad person.

Q. How did this village become infected by Ebola?

KK - One of our sisters (Mrs. HB, wife of MB) staying in Village 1D whose husband was the late Mr. MB is a daughter of this town. Her elder sister (WL) told us that HB`s husband is seriously sick, so we (KK, late ML, mother of Mrs. HB), FK [female], SL [male] and others decided to pay HB a visit as custom demands at her place on the highway.

We also went as team from Village KF and its environs for the funeral of MB. Late MB was a very caring person who showed us much love as is expected from an in-law.

Q. Were there people from this village that went to stay with HB during the time she was caring for the sick husband?

GM - Yes, FK (female, 65 yrs), SL (her brother, now Chairman of Survivors in Moyamba District) and AS [female] were the ones that stayed with her until MB died.

After the funeral, they all returned to the village. Two weeks later FK started complaining of being sick and was cared for by her daughters (KK and MK – both survivors) until the town chief who was also the contact tracer called for the ambulance to come for her to took her to the [Moyamba Holding] Centre. The chief advised all of us to stop visiting houses.

FK died in August 2014 at the holding centre in Moyamba. It was not too long after that the daughters (KK and MK) who took care of her got sick too and were also taken to the same holding centre were their late mother was in Moyamba. Within the same week their children (MS, female, 18 yrs) and LD (female, 19 yrs) got seriously attacked by fever and were carried to the treatment centre (ETC) at Bo. Unfortunately, both died.

Futhermore, the house of SL was quarantined. Because of the elderly people (Alhaji BL, father of HB) and ML (her mother) staying at Village 1D with HB decided to move from Village 1D to village KF because they were also not feeling bright. One day after, the Councillor came to the chief and told him this information, so the chief and the councillor decided to go direct to the house and verify the claim and it was proved to be true. They met these people sick and trying to manage the situation among themselves, so the authorities decided to call on the ambulance to come and take them to the treatment centre in Kailahun in August, 2014; it was unfortunate that the mother (ML) died. SL and the father returned as survivors to Village 1D.

There was another man (BK, 35 yrs) who also came from village KF and assisting in selling medicines in the pharmacy also got infected and died, as rumour was surrounding his sickness that he did the treatment of DS from Village 1C in July. BK lost three of his children (one girl and 2 boys - M and K) that were also staying with him.

In total, village KF had 3 survivors (KK, female, MK, female – who both stay in village KF and SL who sells at Village 1D and is also the chairman of the survivors).

**Interview with MK (female, 29 yrs) – survivor in village KF**

Q. How did you became a survivor?

The late FK (female) who brought the sickness from Village 1D, and who later was taken and died at the treatment centre, is my grandmother. We used to sleep on the same bed unto the period she became sick. As a grandchild who was close to her, I was much involved in taking care of her with her daughter (KK, a survivor too) who happens to be my mother.

After her death, we were quarantined for 21 days. On the 15^th^ day I got sick for three days and on the 20^th^ day an Ambulance was called to take me and my daughter (EG - age 11 months, who died at the treatment centre in Kenema in October, 2014) to the holding centre in Moyamba, and was then transferred to Kenema [ETC].

I left my son LM age 10, who by the grace of God nothing happened to him to date. In total we all in that house were up to 10 persons. We had 8 deaths.

1. FK – my grandmother
2. EG – my daughter
3. ES - my elder sister`s daughter
4. BK - my uncle, who was assisting MB in selling at Village 1D
5. LD (11 yrs) – my elder brother`s daughter
6. MK (17 yrs) – son of late BK
7. FK (13 yrs) – my younger sister
8. WK (14 yrs) – my younger brother

2 Survivors left in this house are:

1. MK (myself)
2. KK – my mother

I am kindly asking the government to assist our children’s schooling.

Furthermore, I am aggrieved with the chief (AM) who was acting as the Contact Tracer then.

Q. Why?

This was the man who made my daughter (EG) to die [crying]. When I got sick, my daughter was OK with no trace of sickness in her. The ambulance came for me to be carried, thinking I would have gone and left my child, but the so-called chief passed verdict that my child should to taken with me to the holding centre which was full of nasty [things] that I don’t wish even my enemy to be sent to. The place was so bad for humans that my child later got affected with the odour, that we were again transferred to the Kenema Ebola Treatment Centre, where she finally passed on.

**Interview with SPL, male, 43 yrs of Village KF, a petty trader**

I am a son from Village KF and Mrs. HB is my sister, who made me to come and I have not returned to date. I am a graduate from SLOIC in Masonry (1995-1997). I was known by my family as a mason, who builds houses. I went to do some work in Liberia for a year and later returned and settled in Bo. When my sister needed to put up structures for themselves (her and the husband, MB), she decided to call me to work on the building project and since then I decided to stay. I have been here more than eight years now and busy in helping my immediate family put up structures in village KF where I used to sleep at times.

During the sickness period, I used to be in close contact with the family as an elder brother who was closed then. The late MB used to suffer a [gall bladder?] pain before, that affected him during the war, so all we could do was to take him to Freetown and later return with him to care for him.

In July, 2014, Dr. BK told us from Moyamba about the serious issue of Ebola and we must be very careful as of that time the sickness was already in Village 1A and MB went for operation (cyst) in August 2014 in Freetown, but later returned and died on 4^th^ September, 2014.

When MB returned, many including me, Imams, Pastors, friends and relatives came to visit him. After after MB’s death a team from the Catholic Church called the Knights of Saint John came for his burial, and they never allowed any family member to be part of them. After the funeral the chlorine sprayers came to spray. The family refused to spray the chlorine but given that they were coming from the Government side they just allowed them to do the spraying. After four days several people (SL, male 30+, MB Jr., male, 11yrs, UBK, male, 35yrs, MB, female, 7 yrs, AK(K), male, 7yrs, FK, female, 6 yrs, JB male, 24yrs, IL (male) and T.) started to become sick, claiming it to be the chlorine that was sprayed or the excess oil on the food that was given to the children around the 12^th^ September, 2014. It was not long before we heard of T.`s death ( 12^th^ September, 2014) who told his mother (HB) that he slept alongside some stranger with a high temperature and started feeling the same in the morning, followed by UBL, due to hunger in the holding centre in Moyamba on the 13^th^ September, 2014.

My father (Alhaji BL) and mother (late ML) were almost always in that house playing with their grandchildren until the funeral rites were done. Not quite long afterwards they started complaining of pains and cold and decided to go back to the village, so I accompanied them. However, the councillor and some people from the medical team went and collected us all to go to the dirty Moyamba holding centre and we were later transferred to Kenema treatment centre (ETC) on the 14^th^ October, 2014.

I for me (SPL), I got infected because I was sleeping on the same bed with my father and was caring for him. Fortunately for us both, we survived the sickness, though he died later when Ebola was declared over.

As a chairman of the Survivors I am kindly asking for help for the widows and widowers and even the survivors (crying deeply) with food and healthcare. I experienced a bad moment at the holding centre, so dirty and with no care, so the Government should ensure that such should not repeat itself. Let the nurses who survived this sickness be placed in positions of trust in administering treatment because they have once gone through it themselves.

I recommend that the chiefs be supported financially to undertake preventive measures as fast as possible so they can be on the alert as they were doing, and even the local chiefs should be involved in governmental decision making.

**Survivors related to HB in Village 1D**

1. SPL - selling at Village 1D
2. HB – wife of late MB - Village 1D
3. MB - son of late Mr. MB - Village 1D
4. MB - daughter of late Mr. MB, Freetown
5. MB - Freetown
6. AP [male] - Njala
7. KK – Village KF
8. MK [female] – Village KF
9. IN [male] – Village 1D
10. Alhaji IL, later died on the 1^st^ April, 2015.
11. people in all died from the contact in Village 1D.

**Field Notes (GAM), mid June to early August 2017 – arrival of Ebola from two directions?**

MB was a retired Pharmacist/Dispenser. After retirement he embarked on a private medical career especially in Village 1D and beyond. His popularity as a practitioner rested on solid personal achievements. He was member of the District Medical/Pharmacy board, a member of various sodalities, such as a (Masonic) *Lodge,* the *Wonde* *society*, and the *Knights of St. John*. After his retirement from the Ministry of Health he established a private pharmacy at Village 1D where he lived with his wife HB, a qualified CHA (Community Health Assistant).

The ban on all private medical practitioners by government were violated by some practitioners under Ebola. A CHO at Village 1D reported that during the outbreak people kept visiting the privately-run pharmacy managed by MB and his family. This violation resulted in several misunderstandings involving the family of MB. The CHO was conscious that during the outbreak people kept visiting this privately-run pharmacy violating the ban by government. It was apparent, for example, that DS from Village 1C had visited the pharmacy for medication before his demise.

HB reported that her husband suffered joint problems. From time-to-time MB was treated by a Dr. [redacted] in Freetown. MB travelled to Freetown for treatment and returned to Village 1D. Not long after her husband’s return from Freetown news came of the death of Dr. xxxx, apparently from Ebola. The news came just 11 days after MB’s return from Freetown, according to HB. It is thus unclear if the infection of MB with Ebola resulted from his trip to Freetown or the visit for treatment of DS from Village 1C.
