# Supplementary material for: Re-analysing Ebola spread in Sierra Leone: The importance of local social dynamics
Source: PLoS One. 2020 Nov 5;15(11):e0234823. doi: 10.1371/journal.pone.0234823 (PMC7644078; doi:10.1371/journal.pone.0234823)
Supplement: S2 Village — (DOCX) [file pone.0234823.s002.docx]

**S2 Village**

**S2A Village**

**KALIA VILLAGE**

**KAKUA CHIERDOM- BO DISTRICT**

**Attendance 12^th^ June, 2017**

FK

IL

Female (husband died)

Female (survivor)

Female (husband died)

Male (children died)

Male (lost 2 wives)

Female (Mamie Queen)

Female (lost husband and children)

Female (lost her relatives)

Female (Survivor)

Male

Male, representative of the chief

OPENING REMARKS

- Appreciated the community and let the community know how vital they are in this work, their children will access relevant information about the epidemic.
- To know what happened and how the outbreak took place.
- Investigation on the health system and on how it can be improved.
- Express concern and care she feels about the outbreak and the survivors in particular including the orphans.

REP. OF CHIEF

Q. How was your setting before Ebola came into this community?

A. We are willing to tell you everything, no matter who, CID or not, because I am the Town Speaker.

- We heard of Ebola in Kailahun and later we had a three-days lock down which became a disappointment to me as we were not able to pray.
- One of our family members (AA) came from Taninahun, 2 miles away from Jembeh to this community and later got sick. He was taken to a health post at xxxx for treatment but was later discharged without any appropriate information about whatever caused his sickness.
- A week after he died (13^th^ August, 2014)
- He was washed and later buried in this community
- After his burial, sicknesses like malaria, fever and joint pains were reported by his pregnant wife (BB)
- BB, wife of the late AA, became sick and was taken for treatment to the Health Post at Village 2B, and was later discharged due to lack of blood without any cause of Ebola [stated] though she was pregnant.
- Two days later, she died after having had a still birth, followed by [death of] her children (CC, the baby and DD)
- That very day a Medical Team came from Bo and stop everyone from reaching each other in this community.
- We were told that even the nurses that treated her are going to be in quarantine for 21 days.
- EE, a brother of the late AA also died here, and his corpse was not buried until after 3 days by the burial team.
- The dress of the Ebola Burial Team created more fear in people and the community so much that more old people became sick and could not survive.
- The presence of the military, police, and DMO caused shocked to all including me.
- 13 people got seriously sick and were taken to the Bo government Hospital in the name of giving them treatment. To my dismay no treatment was given but [they] just clustered them into a non-caring room, were 5 of them immediately died. It was later heard that there is no bed for admission at the hospital, so they are to be brought back to the community school and quarantined.
- We were in great fear that they have come to poison us with the presence of armed military men. Immediately we lost 3 old women
- Our Honourable [MP] brought 13 beds and food for us, though the food was not given to the victims
- The security restricted our movement.
- 12 days later, 9 bags of rice was given to the community with the population of 210 including children
- The District Council brought the food and the Honourable again helped with Veronica buckets with chlorine
- Chlorine was suffocating [us] and even led to the death of one of our brothers (FF)
- The community created 3 burial teams and [these] were working after the death of every victim
- The [government?] burial team took the corpse and placed [it] in the grave and the community team digs and covers the corpse.
- GG was the only survivor out of the 13 people that was taken to Kailahun [Bo?] and returned
- The signs that we saw in this community were red eyes, vomiting, diarrhoea, headache and fever.
- 2 people that washed the corpse of late JJ also died, but one [other] is still alive to date.
- Nurses were referring patients to Government Hospital instead of the holding centres
- Most of those referrals were escaping back to their homes
- In total we had 37 deaths in this community
- HH died at the Gondama [holding] centre after her mother was asked to return here

Q. Who actually is to be blamed in all these?

A. (Town Speaker) - the only one we blame is the almighty Allah and no one should be blamed.

Q. who is [there] to bring on board if such [a thing] happened again?

- If any such thing happens again we should not involve the security personnel because their presence is irritating and causes panic.

- The way the messages of Ebola were disseminated was too harsh and with much restriction. No encouraging words [were spoken] except when it was coming to the end.

We cannot identify most of the graves in this community due to the mass burial that was taking place.

All we did is to make a monument to serve as a general grave site for all; we did the ceremony with support from Commit and Act.

OUR CRY

To help us with a Health Post in this community as all the other centres are far from us especially the post which we are supposed to attend.

From this village to the nearest PHU is 3 miles

From here to Village 2B is 5 miles

From here to our designated PHU is 7 miles

**THOSE THAT DIED AND WERE NOT BURIED HERE**

1. PK [male] died in Bo Hospital
2. IM [male] in Bo
3. BG [male] in Bo
4. MS [male]
5. YK [male]
6. KS [male]
7. MJ [male]
8. MS [female]
9. BS [female]
10. FK [male] died in Gondama Holding Centre
11. YS [female] died in Gondama Holding Centre

**SURVIVORS WITH CERTIFICATE CENTRE OF SURVIVAL**

1. MK [female] now in Bo Kailahun
2. HJ [female] (former TBA) Kenema
3. MD [female] Kenema
4. BS [female] Kailahun
5. MVG [female] Kailahun
6. MJ [female] Kailahun
7. AG [male] Kailahun

- We have 94 orphans, mostly supported by Commit and Act (Hannah Bockarie), Street Child, WFP, World Vision, and one JL

**S2B Village**

**Health Post at Village 2B**

**Bo District**

**Attendance 14^th^ June, 2017**

1. MCH Aide
2. Nurse (In-Charge of the centre)
3. Nurse

**The In-charge**

- She did her course in 2004-2006
- She started work in centre in 2013 unto date, even during and after the Ebola outbreak.

**Constraints**

- Lack of a well-constructed building for a better health service delivery. We are using an old railway building.
- No safe and clean drinking water
- No standard labour room for delivery. Together with the community started putting up a structure for delivery and admission but we lack finance to continue as of now.
- We only have free health care drugs for pregnant women, children and disabled but not for male adults. Malaria treatment has no boundary but is for all.
- The most common reported sick cases here is malaria and the treatment we give are ACT, Coartem, followed by STIs
- People don’t clean their environment.

**Q. *Why are people not cleaning their environment?***

Most youths complain that the chiefs are not encouraging them to do the cleaning, they would rather clean their immediate surrounding

**Q. Why STIs?**

- Because they don’t make use of condoms to prevent themselves [from unwanted pregnancy].
- Most of the men refuse to come for treatment with their wives when the women are suspected or infected with an STI.
- Teenage pregnancy is also on the increase here, we recently had one difficult case that we were able to handle.
- The adolescent girls are more involved than men

1. **Any Village Development Committee set up to manage this health post?**

- They mostly held meetings and we were able to start this new structure with VDC support and from the PBF funds.
- When drugs are short, they also advocate on our behalf. Do cleaning. Especially the women.

**During the Ebola outbreak**

- We were supplied with hand gloves and chlorine and were asked to concentrate it with 0.5 for hand washing and 0.05 for spraying around to disinfect.
- We were preventing ourselves [from infection] by using hand gloves on every patient
- We also went out to our catchment communities to do sensitisations on the preventive measures against Ebola, also teaching them effective hand washing, staying away from eating bush meat and the use of chlorine where necessary.
- We supplied the community with soap.
- We had no Ebola victim from our own catchment communities.

**Village 2A**

- Village 2A is not one of our catchment communities. This community is directly under the Health Post in xxxx and so it had been working.
- In time back, bed nets were to be supplied to Village 2A from the xxxx Health Post but it was not enough. Village 2A was left out and a Sister from Bo Hospital called and asked whether I had bed nets to support the other catchment communities and fortunately for them we were able to supply Village 2A with the remaining bed nets we had. Since then people including the chief in Village 2A refer patients to this centre.
- However, AA, who was the Ebola victim whose husband (late BB) died and was buried in Village 2A came for treatment (24^th^ August, 2014) with one of the TBAs (CC) from their village reporting sick of fever and side pain; she was pregnant.
- She was dressed in white apparel mourning the death of her late husband.
- We actually didn’t notice any sign or [had any] knowledge of Ebola reaching Village 2A, but we had heard of cases in Taninahun behind Jembeh on the highway.
- She was treated for a lower respiratory tract infection with Ampicillin and Amoxicillin). We checked her pregnancy and she returned to her community.
- Our instinct was to use double gloves with much chlorine, different from the way we used to give treatment to patients. We heard of the outbreak in Yamandu from the radio and from a visit by our bosses in May, 2014. Most of the gloves had pin holes which was why we doubled them on treatment. We had training from IRC about the wearing of gloves and the mixing of chlorine before Ebola reached Bo district.
- On the 27^th^ August, 2014, one Mr. DD came and reported the death of AA. And the chief asked the community to go through Sanitary to have a death certificate. All this happened 14 days after the death of her husband (BB).
- We had no knowledge of the [infected] man from Taninahun going to Village 2A.
- Her death was reported in Bo and the burial team went with the DHMT [to do] the burial. After the burial, the team came and quarantined us, as they were told the woman came here for treatment before her death.
- 28^th^ August, 2014 we (the health-centre staff, not the community) were quarantined for 21 days
- I was really not happy with the way we were quarantined, as if we were not doing good work before.
- We hated everything about Ebola, so much so that I (Nurse 2) didn’t want to listen to any radio or hear the sound of ambulance (sirens).
- Feeding was given from the DHMT. Red Cross gave us rice, oil, beans and “blended” with a card.
- Police and the military were too harsh with us, so much that I wanted to give up as a nurse. The community people did not abandon us. They helped us with the leaves for feeding [making sauce] and with water to cook.
- Nurse 2 became more afraid with the guns in the hands of the security personnel.
- We were two and everyone was in their own apartment
- Later IRC trained us on mixing of chlorine and giving sensitization messages to the communities and monitored us and supplied us with boots, buckets, PPE, gloves and chlorine.
- Red Cross also brought food after the quarantine period

**Catchment**

We serve a population of 8367

Children under one (4%) 335

Children under five (17.7%) 1481

Women of child-bearing age (22.2%) 1857

Pregnant women (4.4%) 368

**Outreach points:**

1. J.
2. G.
3. K.
4. Gb.
   - Distances to G. and J. are up to 6 miles
   - We cross streams to locate some of our communities
   - Posters are given to us by Mr. EE from Health Education in Bo
   - We give burial permits not death certificates

**Contact with ETC**

- One of the white people, a leader from the ETC came and told us we are welcome to visit the centre at any time so we can learn the treatment on-going in their centre
- I decided to visit alone as the other worker was afraid then; however, I was refused entrance by the security man at the gate. Then I returned and explained the same to my colleague as well, and with that we decided not to go close to that centre anymore
- MSF was the key player at that centre
- Medical workers were having megaphones telling people not to be afraid of the centre and that nothing will happen to then.
- The Ebola ambulance siren is irritating, and all that was in the mind of the people [as a result] was death.

S2C Village. Village 2C, and Ebola in villages round about

Interview with Community Health Officer

Sometime in early 2014 we went to the monthly in-charges meeting (May

3^rd^ 2014?). There we were told about Ebola which was in the Kailahun district and part of Kenema. We came back to sensitise our communities about this Ebola. Then we placed emphasis on handwashing.

We were on this for a long time, but the community here never believed. They were

saying “it’s all lies and that the government brought the sickness so that when people die they

will not vote in the elections”. We were on this type of argument for a long and told

the chiefs to warn their subjects. If they know of Kailahun today or have families there, [they should take note that] some of the villages are now almost empty, people going back to *sokoihun* [Mende: corners] to avoid the sick and dying. Even in the *sokoihun* people are dying, so I begged then for us all to take some care.

On October 9^th^ 2014, I had a case. This case did not appear initially with Ebola

illness. This man came from the next village about 4 miles away from here with his wife and

he was complaining to be unwell. He is getting fever and also coughing. I started treating

him for acute respiratory infection (ARI).

He was treated for 3 days but the cough never got better. So I had to refer him to the hospital in Village 2C for further investigations. The report from the hospital was worm infestation,

which was treated with albendazole tablets. Before going to hospital, I had already given 3

litres IV drips. The day he came he was with his son, H.

I - Which year?

R - H came on the 15th October 2014. H was complaining of being unwell; he said he had fever and generalised body pains. On examining him, I enquired about the type of

work he was engaged in. He said he was a miner at M. Do you sleep at M.? He

said he only goes to work and returns to his village.

I decided to give him drugs but when I enquired whether he has eaten and if he was

Vomiting he said no vomiting. ORS was already mixed and I gave him some to drink. The

first sip from the cup was never swallowed. He went close to the window and I suspected he

was about to vomit so I went off. He vomited through the window of the office. “Did you not

tell me that you are not vomiting?” I asked. I still went ahead to give him the drugs. I gave

him some other tablets but never gave any injection. I gave ORS to take home and to

return the next day.

After dealing with this particular patient (H), PE’s wife – F - was lying down very weak. I said to her “F, you came with a sick person but you are just lying down weak and sleepy, why?” She said these words “I am not well myself”. Did you take any medicine yesterday? She said “the nurse gave me drugs and injection”. The next day they came with a grandson, MH on 16^th^ October 2014. I asked H if they all lived in the same house. He said “yes”. “How many of you?” He said “six”. H himself, two under-fives children, his wife, and Pa P and the wife. So you are six in number? He said yes now the pa is sick, your mother is sick and you are also sick, the two children are sick. Where have you travelled these

past days? He said they had been nowhere.

In town here, where are you staying? He said with one LM, a school teacher. I then sent my porter to invite the town chief to the clinic. I requested the town chief to ask these people whether they have travelled anywhere before this time. This is because all of them are sick and living in the same house. There is only one that is not sick, H’s wife. So I may want to know what was happening. Now people enquired and they opened up and said they went to T [town near Bo] for one week but returned more than two months now. I said ok I have heard you, but today you are going back to your village. When I am off in the afternoon, I will come to the village with some other drugs. I didn’t want to tell then they were suspects

while they were still at the centre and call the ambulance as this will create more fear.

They easily agreed and went to the village on the same 16th October 2015 [correctly, 2014].

After work, I called on my contact tracer, DF - he was appointed by IRC. We went

there to the village and made our way direct to the town chief. Chief, please chief we have come to you for some information.

I - What is the name of the town chief?

R - JG, JK

[Voices ----------]

I - asked him [chief] if he is aware of the illness in the country. If you give me good information you will be safe, but if you don’t give me good information, they are going to

quarantine the whole village. You know you people are now harvesting rice, not so? He said

“*kpuo - tonya mia bi dia*” [it is true]. I then enquired from him if he can tell anything about P? He said “yes”. He also said he was the one that pushed P to go to meet me. He said P and his wife left this place and his wife left this place to go to T [town near Bo] to attend a funeral. His wife’s sister is married at T. The husband of her sister had died and they went to attend the funeral. They spent over a week at T. Upon their return, news again came about the death of the sister. They had to go back to T to again attend the funeral. This time round, they spent more than a week, they stayed about two weeks. P returned to this village already ill. He has been spending time indoors. I noticed they have been buying drugs and treating him in the house. These past days, I had to ask the wife the whereabouts of P. The wife then told me P was in the house and he is seriously ill. Why don’t you take him to Mr B, the in-charge for treatment - if you fail to do that now, I will inform him myself. It was based on the town chief’s advice they took an okada [bike taxi] to come to the centre.

On that same day I called for the ambulance informing the office about suspected Ebola

cases. The vehicle came the next day to collect then. There was one Ugandan doctor, M, who addressed the community before collecting the suspected cases. While these cases were taken away, one of the under-fives who was before this time taken to Village D [hospital] and was referred to Freetown. This particular baby died at the triage in Freetown. The boy I last saw in the clinic also died on the same day. It was H, Pa P and F - the three people that were collected by the ambulance. On the following Saturday/Sunday I went to Bo. It was on Monday when the office called me – by GS, the DSO. He said those cases you sent, one is positive for Ebola. Entering the office, everybody was moving backwards - going off from me. Then G said to me that if you are greeting people and they are moving off from you don’t you know that people are afraid of you? What do you want to do now? The people are now at the treatment centre at Bandajuma [Bo]. I requested to be tested before moving from Bo. S said they will do that when I start showing signs. OK - that same day (Monday) we were to go back to the community.

I - Where did you to go?

R – To Y - with Doctor M [the Ugandan]. We came to the village [K] and checked records and started line-listing. Then we went to L’s house at K where P and the others stayed.

I enquired about the whereabouts of L. I was told he went to the farm but later

he was found in one of the rooms- not well. We returned to Y to put the quarantine processes in order, the baby that died was not buried until the other day, Wednesday. We then returned to K. I was then requested to be quarantined. I was finally quarantined at my residence at K. I was there for the period of the quarantine. I was quarantined with my porter. WHH

brought food supply. My nurse was not found at the centre when I returned from Bo. So she

was never quarantined. My porter and members of his family where in one house close to

my own house. While in the quarantine house, one Mr. A, a friend of mine, met me at the house complaining of dizziness and not feeling bright. I told him where I am now, I can’t do anything. So, go to the clinic (CHP). I can’t leave this place for now. He went to the

clinic to meet S (the nurse) and S gave him ACT. Mr AK took the drugs

for three days. On the third day of the drug administration he came back to me to still

complain again that he was not well. There was still some dizziness. I told him to go

and see the nurse and explain to her. She will know what to do further.

The nurse advised AK to go to hospital [Village 2C]. She wrote a referral letter for

him but he never went. He went back to his house and after about two days, he realised his

case was now serious. There is a clinic after S, close to B ---[Voices: N G]

R - Not G, there’s a name for that clinic. That is where he went. His wife who was just

seated here by the name of K, her brother runs that clinic. He wanted to see him. He was

seen and treated with some drugs. From that clinic to S Mr. A got tired and sat down in a house – on the right-hand side just at the entrance of S. Somebody met him on her way to Bo. She met him there and gave him transport to return to K. He then took bike [taxi] and returned to K. He was asked on his return to K whether he went to Village 2C. But he said he has been tested and given drugs. A day after, Mr. A himself told his family members to call on the ambulance, expressing that the sickness was now serious with him. That time round I was now in Bo. I left immediately after the quarantine period [ended]. People who were with Mr. A actually realised that the Ebola was real. They noticed that his eyes were red like fire. He was weak and toileting with blood. He cannot even move a few steps by himself. The next day, (on a Sunday) the ambulance came, and he was taken away. He died that Sunday night.

On Monday morning I was told at the office that an Imam brought from K had died

from Ebola overnight.

During his second visit to me while I was still in the quarantine home, he [A] told me Mr.

L is dead.

I - That was A telling you about the death of Mr. L?

R - Yes - he told me. But I told him not to touch the corpse of Mr. L. He said he will

not even endeavour to touch the corpse. Some people can be responsible for

their own death. This man when L was still sick, his brothers met A and requested

him to bathe Mr. L, expressing to him that he has not taken a bath for some days and he

is a Muslim. He was the one that bathed Mr. L.

I - Before death?

R- Before death. When he was asked about that he never gave any clear-cut answer.

Indeed, before I was quarantined, A was my best friend. I was now wondering when

he contracted the infection. The day he was going to that clinic after S - I can still not get the name of that clinic - he went with his daughter called I, a nursing mother. She was the one that supported him on the back when returning back to K. That same day, I confessed

to her people that she was having severe waist pains. She explained to people that she was fighting overnight. She fought with one man by the name M.

I - Did she mention his name

R- Yes.... She called the name of the Pa M. (This particular man hit her heavily in

the dream). I said, yes, that was how Ebola entered into the Kailahun district. People took it

to be a witchcraft issue; some said it was a political issue. I told people to tell her to come to the clinic, but somebody said she cannot even walk. I decided not to call ambulance until I visited her. It was not too long after this that hospital at Village 2C called me to be informed about the death of an under-five child. The close relatives had already abandoned the corpse. While I was being told about the death of the child, the burial team from Bo was already informed. They collected the corpse from Village 2C and brought it to K for burial. Upon their arrival, I directed the team to the house where the relatives stayed.

While preparations were been made for the burial, WHO experts came in my absence - these were Japanese. They stopped at A’s place. The people were now interviewing I. I suggested to them that I should be tested; she is supposed to be a suspect because she has been living together with Ebola positive patients. They said OK – so an ambulance was sent on the following day. Her child was not taken away that day; instead the child was left with a lady, K. The child was later requested to be taken to the treatment centre [ETC] so K took her to Bo. K returned to K after two days and there was another plan to send another person to take care of the child. While on these arrangements, the child died in Bo and we were immediately informed. So nobody left again for any care. The next village called M, on the way to B. A case was identified in my absence. I was in Bo when a lady called MK contracted the infection in Bo, came to M.

I – T [town near Bo]?

R - T again. That family in T that was affected had an extended family in Bo

Town. New Site area you heard about; a house that was at a spot in the New Site area.

I - Is it the same connection? This was responsible for the death of B?

R- Fine...... so that was the area where M attended a burial.

I - New site - that was B Road, Gari-spot?

R- B road - they brought that woman here, but I was not there. Nobody attended to

her. She was referred to hospital [Village 2C] immediately. It was their own ambulance that was used to take her to Bo. She died the same day. The contact tracer was really beaten when he told the relations that MK died of Ebola. He was abused seriously. The relations said they know the woman to be a pressure patient. How can she be an Ebola patient?

Other workers came from Village 2C to tell the relatives the real cause of the death.

The reason for their denial was as follows - a member of the family is a worker in the Bo

hospital. This particular individual by the name of K put away the test result (put it

away). When the staff of the hospital in Village 2C got the wind of the confusion over cause of death of the woman, they went to Bo, including the CHO, to look out for the test result. They found out K had actually hidden away the woman’s result. The Ugandan doctor contacted us to find out why these types of things are happening in my catchment area.

Definitely, I told him the people are very difficult to deal with. I told him further that I was in

the quarantine home, the assigned officers were always drunk. They can’t control the

people. Perhaps for military officers to be sent instead of police officers. This was what was

done and things started to become better.

One of the military officers sent was so vigilant over attempts to go out; he was always there

to enquire whenever anyone opens a door. This particular military officer was preventing

meetings, especially at night. Gamblers were prevented from gathering together. He usually

tells people that he makes sure he puts everything in place, just like what happened

during the rebel war. The Ebola fight must come to pass, he used to tell people.

Everybody was after that involved in the fight. The PC came here with the ….

foundation, they were all involved. When MK died at M a lot of people from here attended her funeral - a large number of people went there during the funeral of late MK’s sister called B. She also died. When she was sick, her children called us, but I told them I was going to call for the ambulance.

Again, sometime when the ambulance is called it can take a day on two before it is available.

Some other times it can be available immediately. The ambulance delayed to come to collect

B. It was her corpse that was met at the time the ambulance arrived. The same K family have an extension at K [speaker’s village]. One of the family members here by the name of M also died.

I - Yes, where again?

R - The same M, a TBA called MM, who came to attend a meeting appeared

very weak. I had to enquire from her about her apparent weakness. She responded that this

is how she is feeling these days. I further required from her to explain to me whether she is

sick - because I was about leaving for a meeting in Bo. She told me to go and if there is any

thing she will contact S, the MCH Aid. The following day she asked S to call on the

ambulance. She was taken to Bo in the evening of that day. She later died at the triage in Bo.

She had two grandchildren. Both of than died after two days. So, this is the total number of

people that died - 18 cases, 6 survivors and 12 deaths.

This same TBA MM; in house there was a teacher, LM - he (a CHW) complained having a boil on the knee; it was the police that came to tell me. I told him to come to meet me if he is not well. I threatened calling the ambulance if he failed to come. In that same house, MM has died and her two grand-children - so this teacher must have had contacts with the deceased. I called the ambulance out; he just met the ambulance that morning he arrived at the clinic. He was then told to go to Bo for a test. His uncle is the one I stay with - the Pa had he told me that the teacher is his brother’s son - you are now sending him to be killed? I told him no - let him go for cure, if he stays in the house he will die. If he goes and people start to see him and treat him, he will get well. It took up to a week without talking to me. After another one week, S was escorted back to K.

I - He was well?

R - He was well. Today he is working with GOAL, having his own living. He added to the

survivors, making a total of six, the other one is M.

I - For the survivors - how many females?

R – Survivors? Chief M’s wife, JM survived, MM, FE. (Voice - there were three woman)

R - Three woman. M, J and FE. These are the three women. Male – 3, female – 3.

R - The home I was quarantined in, people were saying that I brought the Ebola here.

S2D Village

Chiefdom supervisor: [opening welcome remarks]

Respondent: my name is NK.

Researcher: Mummy N

Respondent: yes

Researcher: did the Ebola reach here?

Respondent: yes, it did.

Researcher: how did it get to this place?

Respondent: what brought Ebola here was that there was a man in this town called MM, he was the first person who fell sick. They took him all over the place, but he eventually died. So, when they took him for check-up, then we were told that it was Ebola that had killed him. So, they came, buried him and returned. They came back only to say that whosoever this Ebola disease kills the house of that person will be quarantined. So, he was the first person to be quarantined. They quarantined his relatives too. Second was GW [female], my younger one. She was sick, we took her around and an operation was done on her. So, it was in the course that the disease became worsened. They had to bring her to this hospital. They came from Village 2D and brought her to this hospital. When they checked her it was then they said that this disease which has stuck this lady is Ebola. However, that we did not know the disease, we kept denying the fact. It was when we had gone all out in vain, it was then that the disease became really worse. We started distancing her a little because we were told that when this disease has struck someone, you need to isolate the person, but we were still going there because we didn’t know the disease. We kept visiting her till she died. When she died, they came with a bereavement purse [financial contribution] and they enquired about GWs’ relatives? They said GW had Ebola. Then we asked what it was, because we didn’t know anything. So, they said we were going to be quarantined for a while for them to tell. They left people who observed us. So that is what I know about [how] the Ebola that came to this town, Village 2D.

Respondent: After that they quarantined her children, GWs’ daughters. They took three of her children on the grounds that they had Ebola. The children survived. They are readily [now] with me, three of them. It was during that period that our mum also fell ill. When she did, they took her away. She even died. She died in Bandajuma [ETC]. We did not even see her grave. That was followed by the death of our baby, a toddler, for which we were again quarantined. The very same house.

Respondent: we did not even see the grave. Furthermore, her daughter fell ill and died. They quarantined the house again. They said it was Ebola that had killed the child.

Researcher: It occurred consecutively?

Respondent: yes, three times. Well if that is it, the Ebola, the way in which the Ebola occurred, no sooner they ask you, the rest you will explain.

Respondent: well if it were so, then my own child (voices: everybody talks). So, it was during the course that my daughter, a pregnant woman, she said she had a headache. Is it your head that is aching? She said yes. So I had to come over. Her name is SK. So, I took her to the hospital. We stayed all day, she was treated by nurse M and we went back. When we had returned, at night, she said her stomach was burning. She said her stomach was paining excruciatingly. Oh! You said your stomach is paining? She said yes. So, I said well let me heat up some coffee for you to drink. But that will not be fine because your stomach is like on fire. ...then I saw the condition was getting closer for her to deliver, ok.

Researcher: yes.

Respondent: so, we had to rush... (then she had not reached maturity)

Researcher: then she had not reached maturity or time to deliver?

Respondent: yes, she had not reached maturity. Her pregnancy was just eight months then.

Researcher: ok.

Respondent: ok, so I had to rush and take her...so I had to call [nurse] M2 and I said M2, that patient I brought in the afternoon, whom you treated, complains of a pain in the stomach. So I said here is she, I have brought her back. There were signs of readiness to deliver. There was a nurse, M3, so the woman called M3. Madam M3 and I did the delivery. As she was about to deliver, she vomited. As soon as she threw up, she said she has Ebola. Really? She said “yes”. The vomiting she did. Does she have Ebola? I said well I don’t know anything about this. Is there a law that when a pregnant woman wants to give birth, she must neither vomit nor shit? So, I said if this woman must give birth, if must be dead and indeed the baby was pale and dead. I called Madam M3 who wrapped her. I told her to administer a drip to the lady for whatever cost because she is my co-worker.

Researcher: are you a traditional birth attendant/do you deliver?

Respondent: yes. There is my nurse seated.

Researcher: alright.

Respondent: so, I said we administer a drip to this child for her to survive. I said please. And she said no, she will not administer any drip to her. She said they were going to admit the lady in Bo. So, I said is it so? She said “yes” and she took her off the bed and laid her on the floor. Not too long [after], she took her outside the hospital through the back door there. The patient spread a cloth on the floor and lay there on the floor. She laid there for sometime - even my colleagues from.... saw her as they passed by. They pitied her as they cried in sympathy. I cried till I got tired. My eyes were reddened and swollen. I asked them to help me take her up. This is her brother/sister. The other lady is out there, they have gone to work. We took her up and laid her under that lemon tree at the back of that house and they went and bought her cooked rice flour. They bought the flour; she took it herself and drank. It was during which they said the vehicle is coming. And the vehicle came.

Researcher: which vehicle?

Respondent: the vehicle which used to carry them, the Ebola vehicle, (voices: “the ambulance”). Oh! Yes. They put her in and took them away. I said to myself, if it is so, then I need to chase [follow] her. I went along with her mother. I told her not to sit by. I said let us follow her. We went. We met her lying, sorry we met madam T. lying. She was in the vehicle. So, I said my people, is it that S. [that] had died in the vehicle such that you are unable to remove her? So, I wanted to rush at the vehicle, but they held me up [stopped me]. So, they opened the door to this very vehicle, she came down and spread the wrapper she had on the floor and laid there. I gave her the water I had and another pregnant woman who was around asked what had happened to her. She accordingly explained. Was there a nurse? She said there was. She said why didn’t they administer a drip to you? And I said I did tell my nurse to administer a drip to this woman [but] she refused on the grounds that they will be admitting her in the hospital. She gave money [with] which they bought some medicines at a pharmacy. She started talking clearly such that I could hear her. So crowded it was here and there. There we were till there [it] was light. As soon as light came, they asked us out of the room. Oh! Didn’t M2 say they were going to admit this woman in the hospital? We went out. The things I brought with me thinking that we were going to be admitted, I took out. We slept in the [vehicle] park. Very early in the morning, the lady and I came back, but we were stopped from entering the hospital. They said we would not be allowed access to those people except after several hours. Our relatives kept calling on us to return. So that was what we encountered.

Respondent 2: I too want to talk.

Researcher: alright.

Respondent 2: the child died.

Researcher: it died here?

Respondent 2: yes, the child died here. (voices: “the child died in Bandajuma")

Researcher: Ok.

Respondent 2: the child died on the same day. The mother died too.

Respondent 3: I want to talk too.

Researcher: when she said they chased the vehicle...

Respondent 3: we were on a motor bike behind the vehicle. My brother and I were chasing it. (voice: “they were on a bike”.)

Researcher: thanks

Respondent 3: yeah.

Researcher: what is your name?

Respondent 3: my name is GK [female]. My sister was the town chief, her name is TK. She was here and she had a headache for just three days. She said Y, accompany me to the clinic, this headache is bothering me. Before she had the headache, they invited them to town B to a meeting and she went to that meeting.

Researcher: In town B?

Respondent 3: in town B. They… the chiefs. When she went to that meeting, she slept there and when she was back, her eyes were very red and her head was aching seriously. When she was back, I asked her “Sister T, why are your eyes so red?”. She said she had headache all night in the room she slept. She said she had smelled chlorine all night. This headache is serious. So, she asked that I accompany her to the hospital. The two of us went to M2 and she treated her. It was in the morning. We returned. Upon our return, she did not improve so we had to go back to the ward and then she, M2, said that she is referring sister T, so she could be transferred to Village 2D. When she had received the referral document, her colleague chief at Village 2C, so I called him, Mr. S, and I explained to him that sister T is ill, that she cannot travel on bike. So, I want you to come along with your vehicle so that you can help take her to Village 2C. I opted to buy the fuel for her to be taken to Village 2C. The man came very early in the morning. The man came and sister T was put on board. That lady had a child then. I asked A to follow her, who is an old person and they went. M2 contacted her colleague nurse at Village 2C. When sister T was at the gate, the said “that patient out there is an Ebola patient. Don’t attend to her”. Those of us here have Village 2C as our home and somebody hurriedly left the ward and came to us from K. They started pushing us off. They said we should not park our vehicle here. The person came and realized that it was sister T. So the person called me at once and told me that such is the condition. And I said ok. At that time I was confused, I didn’t know what to do. They came and she said, “Oh! Y, they said they are taking me to Bo. They are taking us to Bandajuma.” (crying) ‘*”it was painful seeing my sister leaving me’’.* She said I must not forget the children. When the vehicle arrived, sister T was the first person they asked for. In fact, it was for her that they sent the vehicle for. They took everybody along, so I had to call my brother/sister and explained to him/her. They stay in Bo, so I called they and told them that they have taken sister T to Bo. They went and met them there. They met them at the hospital. They were not allowed to get closer to them.

Researcher: Bandajuma [ETC]?

Respondent 3: Not yet Bandajuma, but Bo. Except when my son had to try in. He is SM.

She said she wants to drink, and he bought her a pint of soft drink and a tin of milk which he mixed up. When they realized that there was an influx of people, they lull them to forget and early in the morning when my son got there, he called to say that they didn’t see sister T at the hospital, that they have been taken to Bandajuma. So I called SM and explained to him that they have taken sister T to Bandajuma. SM made several efforts to see her but to no avail. So he bought a phone and recharged it and gave it to the man at the gate, instructing him to give to her aunt who is TK, for us to at least talk so that I can hear her voice. They took the phone to her and the phone rang several times but sister T couldn’t pick up. So SM called me and said that he does not think his aunt is alive. He said the phone has rung too long but she has not picked up. He is a Mandingo by tribe. They took them on the nineteenth...sorry the ninth of December but died on the nineteenth. It was after ten days. The child used to go there [ETC] frequently because he/she was known by the whites. The white people came and said that the chief whom we took along few day ago has died, but we are going to call her son, SM, who often visited her. We wanted him to take a look at his mother for him to confirm whether it is she. All of us were unable to go but SM said they went, the body bag was brought out, unzipped the bag down to her belly and they asked him whether that was the mother. He answered “yes” and they took her to CKC [cemetery] where she was buried.

Researcher: Thank you. As we started, we have heard the information. We have seen what has occurred and I don’t think we can easily forget.

Respondent: forever, we will not forget.

Researcher: we will not forget it forever. It has made us to know what to do when there is a recurrence of such events. Many things went wrong.

Respondent: many things went wrong.

Researcher: so I thank you.

Respondent: ok.

Researcher: is there anybody who wants to....ok.

Respondent 4: we were the first to lose a relative. The child was called M [young man]. For a very long time now we have been here and we knew he had tuberculosis and a nurse used to come over to treat him and she transferred him to S [health centre] and he went. He used to go there. When he died, when the illness got worse, we went to the nurse and reminded the nurse that this man had TB. The disease has become really serious. She knew that he had died, so we came back and told her that the [young] man had died and that we had buried him. We returned and his death was described as Ebola. She said that “the deceased was my patient, he had no Ebola. Now that you have said he had Ebola, I don’t have an idea about that”. So we went to the farm and spent all day there. As soon as we returned in the evening, we were all seated and everybody was stopped from moving. We were about forty. Am sure it is on paper. So they quarantined us. It was during the time the soldiers and the white women came. The soldiers had their guns placed on their shoulders. We were the first. This town fled in fear. Everybody was scared of the soldiers particularly the way they entered this town. Even the pupils were scared off. Some people sought hiding in the coffee plantation at the back of our houses. So we are the first people who contacted Ebola in this town. So that was it.

Researcher: sir, please wait. You said soldiers and police officers came in carrying guns on their shoulders?

Respondent 4: they carried cameras on their shoulders.

Researcher: when you saw the soldiers and the police, what came to mind?

Respondent: that because we have been quarantined that [was] the reason for which the soldiers have been brought so that we will be stopped from moving. They immediately told us not to move from where we were. We were barricaded and told that we have been quarantined. We were not to leave the area and police officers were left to enforce it and the soldiers returned. Three soldiers remained and some police officers.

Researcher: what did you say came to mind when you saw the soldiers.

Respondents: (laugh) it reminded us of the war.

Respondent 5: my mother skipped a few things when she was speaking, which I want to add. When the death occurred, we had a meeting ....her fellow nurse

Researcher: who met?

Respondent 5: the chief of B. Mr. K. It was then that M2 admitted that it was she who called the vehicle. She said it was actually she who called the vehicle to come and pick up S. She said she did not call the vehicle for anybody, but it was for S. It was she who called the vehicle to pick up S. (Voice: “let us leave it as that”)

Researcher: but the vehicle actually came?

Respondent 5: yes, the vehicle came and took her away. She said it was she who called the vehicle. It was on that very day she called and the vehicle came and took the away. It was she who actually called the vehicle.

Respondent 6: ...I want to explain. It is also about the Ebola. My name is J. She [S] was eight months pregnant when she had headache and came to this hospital. The nurse questioned her and she called her father and told him that the lady has Ebola because she had been frequenting quarantined houses. So the dad came and said now that this lady is neither vomiting nor having diarrhoea, so how you can tell she has Ebola. I don’t know what happened thereafter. She was sick; she could not attend to nature. Even for her to defecate, we had to get her traditional medicines. Everybody at the house and even those around never saw her vomit. It was during which the vehicle was thrown at him. They said she has Ebola. We were seated when they came for her. They took her away. There was no news about her death. We know nothing.

Researcher: so, since they went with her?

Respondent 6: in the pregnancy. Eight months pregnancy! They took people along as though they were animals. They will just say let us go and bring them. Here at Village 2D.

Researcher: what is your name?

Respondent 6: My name is JK.

Researcher: JK, at the meeting that was held there, madam M2 said you were the one who called the ambulance/vehicle, so that is why.

Respondent 6: yes, she called it.

Researcher: how did the meeting end?

Respondent 6: that meeting ended in chaos. They ended in chaos.

Researcher: apart from that meeting, did they hold another meeting?

Respondent 6: No. By then she [Nurse M2] was not here. She was in Bo. Since she went to Bo she has not returned. They quarrelled a lot because whomsoever got ill and was brought here...

Researcher: what brought about the quarrel?

Respondent 6: the language she used made them to quarrel. The chief [K] said they should not blame anybody that those of them in Village 2D are the ones responsible for the Ebola outbreak. He [K] said she was responsible for the outbreak of Ebola in Village 2D. The very chief of Village 2D.

Researcher: the chief?

Respondent 6: the very chief. He said it was he [chief K] who actually said Ebola must come to Village 2C. He said nobody else said so but he.

Researcher: ok.

Respondent 6: the chief, chief K. He came and held a meeting. When he held that meeting, he said nobody in this town should blame anybody for Ebola, rather we should blame him. He said it was he who brought Ebola to this town and if we were to swear anyone, it should be he. After saying these words, he dropped half a bag of rice, jumped into his car and went away. Our chief, chief K, our chief. Mine is finished. All of mine is finished.

Respondent 7: I, the speaker of the message, my name is BS. I was a health promoter here. (voices: “you wait...”) in the month of November, on the eighth day, there was a man known as JM whom I used to visit every day after work. There was no house between his house and mine. That man went and did brushing but did not complete the clearing. He had stopped going to the bush. Since March, till the man died on the eighth of November, he had stopped going to the bush and I did not see him go to the bush or someone come from a village to visit him. I did not see anybody come from ….. to visit him. The person was sick, he couldn’t even go out to see others. His only brother whom I saw visiting him lives in N. He came, slept and returned. On the eighth of November, as I was seated here, there is a man there they call MP. He came and said that the man called Mr. M. At that time there was a law that when someone dies, somebody from here must call. Upon Mr. M’s return to his house, it was less than two hour when two vehicles came from Bonthe [District] together with two bikes. They met me seated here. They said we have come here today. They said they have seen it in the internet that there is an Ebola patient in this town. Has any death occurred here? A death occurred here yesterday but giving that this Ebola as it is being talked about, is it someone who carries it or brings it? Mr. MM has died over some eight months ago, not on one occasion did i see him go out in the open, neither did I see someone come into him. I said I don’t believe it. Then a bitter argument ensued between us. And they asked “why?” I asked them whether Ebola is something someone brings or carries out? I even did him a referral on two occasions and they did not detect Ebola. Apart from that, there is another man in Village 2C, PA, I suppose he will be able to tell because I did letters...but I said if you had said so, I told them that I do not trust that. So they said we must get to the town, and we went to the town and went to Madam, Madam T, and they did the submission. “Hey!” she exclaimed, “it has been over eight months I have not been seeing that man moving around. He had not been even going to the bush too often. As we were discussing, then troops arrived. They said they were from Bo. They said the man who died yesterday had Ebola. Therefore, those people should not go anywhere. They should now be quarantined. What was more was that there were forty...people in quarantine. There were babies and toddlers for the twenty or more days until the period ended.

Researcher: alright.

Respondent 7: what followed was that after two weeks the troop came here and said the first was seen in the internet but this is the reality, that which they have come to say here. I asked “what is it?” A woman has died here, and that woman is named G. Her death is really Ebola, but the first was not. That which occurred in this town was what prompted the reprisal. I said if that is what they did, then it is up to them. Ehhh, the lady, G, left here on the fifth day for the hospital at Village 2C. When she came here, in less than a few days the woman died. We were seated here, there were people who were in this town. The troops from Bo went straight to the house. They said we have understood that this woman came and died; she is a very popular personality. M, T, L, everybody came to them. Who are those who came sometime ago? It was JS and others who came. I said my friend hold on, the first thing I want to tell you is that Mr. G, do mean to tell us that those of us in this town are not law abiding? Why? They said when someone is sick, and you pay that person a visit, you pay the fine of two hundred and fifty thousand Leones. But are we people who find money but do not know what to do with it? Whosoever told you that told a tale. They said it is true. The two people who said that are very popular personalities. They can’t tell lies. I said you know what I am telling you? They said OK, I said they have told you a lie, whosoever may have told you that told a false story. When we were departing from that town, we said to ourselves that we should keep an eye on each other. Anybody who chooses to go to the house of a sick [person] is an arbiter of the law. Is that why you are saying this kind word? And they said it is true, but that was what they were told. We were together and there was [the] corpse awaiting burial. MF, her corpse was lying out there. We left there for G’s place. We kept moving back and forth. Man say the truth, all of this area is quarantined and what I know is that every morning on my way here, I go through this place. What is fine and what I know is that that woman only stays with her children in that house. So when they went they quarantined six people. We should not go by that. Apart from that, we were here when madam K, as she said earlier, her daughter wanted to give birth, and she brought her here. Mammy, madam T was sick sometime ago, as stated by her sister. The wife of Mr. SN, madam E, who was also sick, but was at her house. The woman miscarried later. I don’t know whether she gave stillbirth or what, I don’t know what happened. I left here together with others for B; and we crossed [met] the ambulance on the way to collect those three people. On which day they took them away from this town, ehhh, they took them away...(voice: “the ninth”) it was on the ninth that they took them away. (voice: “the ninth of December”). Voice: “sister T went on the eighth and died on the nineteenth. We were here when we first heard about the death of MB. It was on the eighteenth of December that we heard that God has taken the life of S. Ehhh, (Voice: “E”). Madam E it was that very day that that God took her life..

Researcher: here or in Bo?

Respondents: Bo. There at Bandajuma [ETC]. It was in Bandajuma. (voice: “when you are taken from here you are carried to Bandajuma). Apart from this, Madam T, died on the twentieth of December. Besides that, following the death of Madam E, six people were quarantined. Ehhh, Madam MB was quarantined for sixteen days. Madam ES had sixteen people quarantined. And for Madam TK, sixty-three people were quarantined. (voice: “she was a town chief”.) Apart from that, there was a child, the child of that woman there; his name is MS. MS died on the twenty eighth of December and six people were quarantined. No, it was eleven people who were quarantined. The one next to his was the woman there on whose account Madam B spoke. Madam G, Madam G, though pregnant, she came here frequently. The person who treated her was M. It was M who saw and tested her. There was a day when her illness got worse as it was rumoured. And they said they want to make a call for the woman. They made the call. On which day did they make the call. It was on the ninth of December that they made the call and they took the woman away. She couldn’t make it when they took her away. Twenty people were quarantined after her death. Following the death of E, her children were struck by the disease. An ambulance was called from Bo and they came. When they came, they took away four of the children from here. When they took them away, A [girl], M [girl], J [girl] and J2 [girl] had no problem. They came back to this town. Apart from that ehhh, AS was also taken from this town in January. It was on the twelfth day that they came for him. When they went they said it is Ebola that is affecting him. They were three in the contact...contact...(researcher: quarantined)

Respondent 8: in quarantine. They came with MS on the twenty fourth of January.

Researcher: did he survive?

Respondent 8: he survived. The survivors in this town amounted to five. (voice: “she did not just survive like that”) the last person ...the last person...am not stopping. He [M5] was the last person to be taken, so we got angry and said he will not go.

Researcher: So you got angry? We said he will not go because you had taken away our people and they are just dying. So we are not going to allow you to take along M5. And they said for this we will bring him back.

Researcher: was that what they said?

Respondent 8: Yes, openly on that street. They said this [one] will recover in God’s name. And he came.

Researcher: Is he here?

Respondent 8: No. He is a stranger and has returned home. Eeeeh, apart from the eight of November, (voice: “we will say whatever we have to say”). At that time MS died with the other three infected, the Ebola ended just as Mummy had explained. Ebola finally ended in this town. That happened on the 2^nd^ January, 2015.

Researcher: January 2015?

Respondent 8: Yes. So on that day, when they went, Mr. P said if there is no sick person, then those people must be freed. That is what I know.

Researcher: OK. Thank you.

Respondent 8: OK.

Researcher: eeeeh, all of what you have said, it seems all of what they have been saying as we have been seated here, we are going to put everything together and document. Having documented it, (clapping) Mr. T said it earlier. Do you know why I asked what happened....(voices: “these two actually took care of us”). I asked a question earlier which is: why is this town called …..? Did you see it right?

Respondents: yes.

Researcher: the youths do not know. It is today they have known. This will be recorded for the youths to know in the future. So they can have it in mind at all time that a disease of this kind occurred. Ok, so that is what we do not want to miss out.

Respondents: ok.

Researcher: so we have come from the university. We are going to document everything (voice: “for it to serve as History”) but it is not documented. And too many diseases have occurred and they took them to the bush. But it was not documented (voice: “not at all”.) At this time this is the reason for which we are here to research and find out the details. So we want it documented. It is not even documented. Maybe they are even telling lies. The documents are mixed up. But this is documented. That is why we are thanking you. Right?

Respondent: ok.

Researcher: thank you.

Respondent 9: after the Ebola, chief K asked that they should quarantine the entire township.

Researcher: ok.

Respondent 9: while in quarantine, they took down our names. If you happen to be at a given house, there they will take your name and quarantine you there. Nobody was allowed to move from one point to the other. Any such attempt will amount to a fine of fifty thousand Leones and some people fell as victim. So chief K came here and said they should quarantine the entire township. He admitted that it was he. He summoned a special meeting at the hut.

Researcher: OK.

Respondent 9: We were ...

Researcher: What’s your name?

Respondent 9: F. We were working with these people. They recruited us to work with them. When they were quarantined we used to oversee them. The man who wrote the note used to oversee them. At some point those of our bosses who came from Bo shouted at us saying: why is it that when things go wrong you don’t call to tell us? They will call us all night. They don’t come to the hospital, rather they go to that man. We will move together all night till God blesses them. When they are quarantined, they come with strange food which we are not used to. So we will tell them to keep it. There are too many things in it. They said it will make you vomit, have frequent stool. So if we are not used to this food let us keep it. Let’s be eating it. When the quarantine has ended, we will eat the white man’s food. Even if you were to go the bush for about a month is nothing bad. So that of Madam T’s business, she is my sister, I have been with her since I came here. It was during which the disease caught her. She was in Village 2C when they called me. She called and asked “F, where are you?”. She said “don’t come! Please don’t come!” she said “wait!” They are coming with Madam T. I went to the gate myself and stood there. While at the gate, they came in with madam T in a vehicle. We stood there with the hope that they were going to take her from the vehicle. Unfortunately, they didn’t even touch the door of the gate. We stood there for long and a heated argument ensued among them. They did not even countenance them. Even the letter they took along was not accepted. They turned the vehicle back and I mounted the bike. The phone they gave mama had fallen some two weeks ago. When I mounted the bike, I went and reported to my boss. They brought pain in our lives but at any rate, we thank God. We thank god.
