# Supplementary material for: Re-analysing Ebola spread in Sierra Leone: The importance of local social dynamics
Source: PLoS One. 2020 Nov 5;15(11):e0234823. doi: 10.1371/journal.pone.0234823 (PMC7644078; doi:10.1371/journal.pone.0234823)
Supplement: S3 Village — (DOCX) [file pone.0234823.s003.docx]

**S3 Village**

**S3A and S3B villages. Field Notes on chain of Ebola infection in Villages 3A and 3B**

**Village 3A - 13/02/2019**

Sharing greetings. Famalui was presented to the community. Which was well received.

To the meeting proper, one of the visitors asked how Ebola entered Niawa Lenga and how they were able to end it when it went on for many months in other areas.

In response the town speaker, SEJ said ‘’one of our daughter was resident in Bo married to a man who was a bike rider born in Kailahun. When the Ebola illness started it was announced that no one should encourage strangers from Kailahun or any other part of the country. She left Bo to the village to witness funeral rites of her grandmother. Unfortunately, when she returned to Bo she met her husband’s brother from Kailahun suffering from Ebola. She then attacked her husband about the issue that no one should lodge a sick or person stranger. Why had he encouraged his brother to come and stay with them? All the other tenants feared and became suspicious of the man. After a week his illness worsened. He was admitted at the Bo Government hospital. While in the hospital our daughter was attending to him but could not make it up, and died after four days.

Two weeks after his death our daughter herself got infected. Her husband went to his in-law (his wife’s aunt) a trader in Bo, and explained to her that her daughter is seriously ill. They decided to take her to Village 3A through Village 3C [see separate note] but died along the road. Her aunt washed her corpse and dressed her for burial and arranged all her burial rites. After a week the aunt herself showed signs of infection. She was a born of Village 3A and paid some boys to take her on hammock to Village 3A, using the same road through Village 3C. But when they came to Village A they just left her on the road leading to the health centre. She then talked to one rider who was fully dressed with wind breakers who boarded her to the health centre. But the sitting councilor (a veteran nurse) told the nurse ‘’don’t touch that woman, I’m suspecting her of being infected by Ebola. To save the community no one should go closer to her’’, the councilor added.

After a few hours the nurse hung drip on her and started attending to her. They sent to her mother in D. She came and went straight to the Health Centre where her daughter was admitted. Both of them got involved as a result of their stubbornness. When the family tried to caution the mother this was what she said: ‘’if my daughter is going to die from Ebola let me also die because I have come closer to”. Her daughter died after four days. An Ebola burial team came to bury her. Her mother, suspecting she is infected, refused her to enter the family house. She later went to D where she was compelled to go to Bo for testing but later died. After two weeks the nurse also got sick. She went to Bo but could not withstand the tension in Bo and came back to the village. While in the village she became worse. An Ebola team came and quarantined the entire hospital and said no one should stay at the hospital.

Pa D offered her one room in his house. She later went to Bo, but after a day she died. Two of Pa D’s children who slept in the room after her death got infected. One died and the other survived. Pa D’s elder son who washed his younger brother’s corpse also became infected and died after a week. The boy in question was in the house of Pa D’s elder brother, where the Ebola entered and caught his uncle’s wife. They were taken to the Ebola Treatment Centre at Bandajuma and later survived. S. who was working closely with the nurse became sick later on and died.

Visitor’s asked how many people died. The answer was five deaths and three survivors (two female and one boy). After E’s death others got sick but survived, one of the elders ended.

Visitors also asked which organizations helped the community. ‘’We got assistance from Catholic Relief services, World Vision, RADA, and others, who gave supplies of various item to survivors. RADA engaged survivors every month to be talking to others. They gave Le 300,000 each to survivors’’, the chief commented.

Visitors further asked whether other villages in the chiefdom got infected. In response the town chief said ‘’there was sick people in Village 3B and another village, K’’. Village K. was very pathetic. Cases in K. emanated from W. [a town near Freetown, the capital]. The husband of one woman and his other wife died of Ebola. The other wife decided to escape with her four children to K. Two of the children died of Ebola. Pregnant, she later became sick. The community asked her and the children out of the community. She ran into the bush with her children, later dying and leaving the two children as Ebola survivors.

Visitors finally asked role the community played and what ended the Ebola in the community. The town speaker responded that ‘’we stayed glued to the bye-laws, curtailed movement, stayed at home and followed the laid down rules of the Government. The community complied and stay within the by-laws and worked with the instructions of the security services in maintaining law and order. For example, two people were not allowed to fetch water at the same time. An Ethiopian doctor came to supervise our quarantine. At first, no proper arrangements were made about feeding. One of our local politicians saw the problem, and brought some supplies, challenging the security to let him enter the village. The authorities then realized the need to organize proper supplies, so the agencies mentioned by the chief came to help. This meant that people did not need to move about hunting for basic supplies.

**SEJ, JY, L, M, AS**

**Village 3B**

16/02/2019

‘’One of our daughters was sick, suffering from tuberculosis. She was taken to Bo Government hospital and admitted in Ward 2. She was accompanied by her mother, Madam S. who was caring for her. While responding to treatment, one of the nurses died. All the patients ran from the ward fearing Ebola. Madam S who was taking care of the sick daughter became very seriously sick and came to the village. Later she died. It was announced that all deaths must be handled by an Ebola burial team. The team was then called upon. When they came, one of our sisters was given PPE so that she could dress up the corpse before burial. The corpse was then laid on pile of sticks to carry it to the cemetery for burial. Why the burial team did not have its own stretcher is a mystery. The boy who carried the sticks away from the cemetery after the burial also got sick. We were expecting that the team would have informed the community whether the first death was an Ebola case. But they only sprayed the room with chlorine and said no one should sleep in the room. They promised that they will inform us later. Because we were in doubt about her death and the team didn’t inform us earlier, people entered the room to sleep and got sick. Even the tuberculosis patient also became sick and died. In all seven people who became sick were taken to Bo Ebola Treatment Centre at Bandajuma. After which the entire village was quarantined. The day they came to collect the sick people to go to Bandajuma one of the patient’s died before they left. In total 10 people died and seven survived. The survivors are still alive’’**.**

**Statement by the town chief GJ. In attendance elders AB, FK and DK.**

**S3C Village. Visit to Village 3C, N. road, Niawa Lenga chiefdom, 18^th^ February 2019**

We (FMK, EYM and PR) visited Village 3C as part of our investigation of the Niawa Lenga chiefdom Ebola infection chain. We were welcomed as the first visitors ever to reach the village by 4-wheeled vehicle. This cannot be true, since the track we followed is an old colonial post road leading up into the hills NE of Bo, but probably they meant “in living memory”. The stick bridges used by okada riders were in very poor condition and we had to watch our progress very carefully, since there were several streams and swamps with deep water even at the peak of the dry season. We held a meeting with the town chief, various elders and about 50 villagers. Women and youth were well represented. We gave the usual *famaloi* to the chief and were granted permission to state the reason for our visit. We explained that we were following up on Ebola infections in the chiefdom and had been told that the index case for Ebola had passed along this road in October 2014, and we wanted to know if this was correct. We were informed that this was the case, and the story was explained. A woman from Bo [name deleted] who originated in the chiefdom had some chronic sickness problems and was in the habit of returning to her birthplace to seek local herbal remedies. On this particular occasion she started to feel ill while traveling and stopped in Village 3C for the night. Strictly, housing a stranger for the night was against Ebola byelaws, but she was too ill to return to Bo and was a daughter of the chiefdom. A blind eye was turned. The chief had no chance to report the incident since by early morning (05.00) she was gone. Overnight, her helpers had arranged a group of young men to carry her by hammock to Village 3A, at first light. The hammock party did not report its departure to the chief, as protocol required. By this it was implied they knew what they were doing in hiding and transporting a potential Ebola case. The woman and her carers discharged the hammock carriers outside Village 3A (perhaps not to draw attention to her arrival, but perhaps also to shield the carriers) and relatives came to help the woman reach her family home. The hammock carriers were said to have had no further health consequences, though they were not available to us for interview, being at work in their farms. There were no further cases of Ebola in Village 3C. The people further explained that relations between them and Village 3A were not very close. They were isolated from Village 3A by some quite high hills – outliers of the main Sierra Leone granite escarpment – and felt neglected. Maybe this explains why no follow up was made by the chiefdom authorities, even though they had earlier told us the story we had just been told. The meeting then re-assembled around our Land Rover for a photograph to mark the occasion of our visit.
