# Supplementary material for: Re-analysing Ebola spread in Sierra Leone: The importance of local social dynamics
Source: PLoS One. 2020 Nov 5;15(11):e0234823. doi: 10.1371/journal.pone.0234823 (PMC7644078; doi:10.1371/journal.pone.0234823)
Supplement: Attachment — Submitted filename: Richards-etal-Response-to-Reviewers_Final.docx [file pone.0234823.s007.docx]

**Re-analysing Ebola spread in Sierra Leone: the importance of local social dynamics (PONE-D-20-06525)**

Dear Mary,

We thank the reviewers for their comments on our paper and are pleased to provide you with our responses below.

We trust the these are to the satisfaction of the editors and we look forward to hearing from you.

Kind regards,

Paul Richards

On behalf of the Ebola Gbalo team.

**Response to Reviewers**

**Review Comments to the Author**

**Reviewer #1**: The authors present an ethnographic study of the transmission of Ebola Virus Disease (EVD) in two districts of Sierra Leone between June – December 2014 with the objective of enhancing understanding of the heterogeneities of disease transmission observed in the extant quantitative epidemiologic data. The study methods and rationale are, in general, adequately described, however a section of the “Limitations of this study” (lines 270-275) is unclear.

***We have reworded this section to make it clearer.***

The narrative of the Results section should more clearly and concisely demonstrate linkages in the chains of transmission.

***We have considered this suggestion in the light of the totality of review comments received and have made a few small changes to enhance clarity, particularly in the Discussion, however, in line with Rev2, we think the richness needs to stay so we have not removed any detail. We also believe the linkages are clearly stated – and Rev2 does not request further clarification.***

This section would be enhanced by a map to demonstrate the geographic relationship of villages to each other, and epidemic curve of cases in the two to provide better context for the ethnographic descriptions.

***We would not be happy to do this, since we have carefully anonymised the villages, since many villagers feel stigmatised, and a map would allow anyone with local knowledge to identify the villages. Similarly, we cannot show the curve for each of the villages, but we do give (in Fig 1) the data for each of the chiefdoms.***

Table 1 is not labelled as such, and Figure 1 could benefit by having all data points labelled.

***Table 1’s title has now been moved above it for clarity. Fig 1 only labels the data points relevant to the areas described in this paper, again for clarity.***

Description of transmission of EVD in Village 3C (lines 628-640) should be enhanced so as to explain the relevant rapid disease control in this location.

***We have made some additions to this description.***

The Discussion and Conclusion are generally sound; however, the authors could make clearer linkages conclusions and the supporting data.

***We have extended the conclusions, and parts of the discussion, in line with specific suggestions from Reviewer 2 and believe the links are now stronger.***

The initial section of 2. Heterogeneities resulting from structural or organizational deficiencies (lines 696-707) is unclear and should be re-worked.

***We have reworked this.***

The overall conclusion (lines 798-807) is well laid out, but given its importance, the authors should elaborate upon their recommendations for the use of ethnographic methods is future outbreaks.

***Thank you for this. We have now elaborated the conclusion on p.16.***

**Reviewer #2**: This is an important article, that makes a significant contribution to our understanding of the West African Ebola outbreak by reconstructing infection chains through rich ethnographic data to help explain local heterogeneity. The article however has the potential to state its contribution even further, and I would encourage the authors to do so more explicitly. As the authors already hint at, they are elucidating the importance of ethnographic perspectives in developing a richer picture and a better understanding of epidemiological data. This has important epistemological implications that extend well beyond the outbreak of Ebola in West Africa.

Indeed, given current debates around evidence in the COVID-19 pandemic, the article offers important reflections on how collaborations between epidemiologists and anthropologists, the combination of qualitative and quantitative data, can produce not only more context-appropriate responses but also more accurate efforts to explain and model an epidemic taking into account social dynamics. What becomes visible through the stories collected seems to me to speak to a larger point about what evidence and whose knowledge counts (and which *should* count) in the ways we come to know epidemics.

Of course the article rightly argues against a one size fits all approach but it would seem to me that the conclusion is not simply 'context matters' or 'every case is different', there are clearly, as the authors state, a number of 'categories of factors' that can help explain local heterogeneity in ways that need to be considered for different localised outbreaks (either in other parts of Sierra Leone, or in other countries and other epidemics).

The broader implications of this approach are already in the text, but in order to state their full potential it might be useful to include a more explicit consideration integrated right from the introduction and then more streamlined through the discussion and conclusion, rather than just a sentence at the end.

***Thank you for these helpful observations.***

***We have added more to the abstract to highlight the explanatory value rich ethnographic data for interpreting and explaining heterogeneity and the importance of integrating this kind of research in epidemic epidemiology. And also at: p.3 just before the Methods; p.6 at the end of Limitations; p.13 in the first part of the discussion; and p.16 in an extended conclusion where we have incorporated some of your language.***

A theoretical reflection on the 'social life of data' and the challenges of integrating this kind of interdisciplinary collaboration and local knowledge into an outbreak response may be beyond the scope of the paper, but could be hinted at as scope for future reflection.

***The point is well-taken and deserves our attention, however, we think (as you also acknowledge) that it would risk overloading the paper if we added it, where a clear conclusion is needed. Nevertheless, we have elaborated the conclusions including reflections on the importance of interdisciplinary research in outbreaks.***

Some smaller points more specific to the text:

- The data is acknowledged to be from the beginning of the response, and there are some interesting reflections on how it might have been affected by different kinds of intervention. Because the data can't speak to this, it would seem premature to suggest that externally driven emergency response is not the most effective (line 647). It would of course be fascinating to have the same level of depth of data across districts and from later on in the response, and this may be possible for a future paper.

***In fact, our data cover the entire period of the response (data were retrospective) but focus mainly on the earlier months because that is when the epidemic took hold in the two study districts. By the time the epidemic ended in the study districts, the national and international responses were in full swing. We agree that data on experiences of later-affected districts could potentially show somewhat different findings. We have qualified the statement in line 647, nevertheless it is true that for early-affected districts before adequate national and international support was leveraged, the external emergency response was limited.***

- A brief note on ethical consideration more directly on the handling of patient data on hand-written logs, test results and the private records of a nurse in Moyamba, would be important

***We pondered the ethical implications of handling this data set. It has been preserved in an XL data set and will be anonymised before it is submitted to the National Ebola Archives. We have been careful not to reveal any personal data of patients. Essentially, the data we have used are equivalent to the data included in the national records created by the National Ebola Response Commission, and now bequeathed to the Njala Ebola Museum and Archive. The Moyamba Holding Centre was a desperate piece of improvisation in which many people died in tragic circumstances. But we felt that the devotion of this nurse in keeping (evidently faithful) records on his own initiative at such a harrowing time deserved some recognition. Over time, people are asking what happened to their relatives during the Ebola epidemic and the existence of this source when added to the NERC records will help answer those heartfelt queries.***

- Does Fogbo (line 384) need to be anonymised?

***Thank you for spotting this – we have now anonymised that reference (to Village 1A).***

***Further journal requirements have been adhered to.***
